# First-Repetition Mechanical Performance Retention During Repeated Bench Press Following Standard- and Cambered-Bar Warm-Ups

**DOI:** 10.3390/jfmk11030286

**Published:** 2026-07-22

**Authors:** Dawid Koźlenia

**Affiliations:** Faculty of Physical Education and Sport, Wroclaw University of Health and Sport Sciences, 51-612 Wroclaw, Poland; dawid.kozlenia@awf.wroc.pl

**Keywords:** resistance training, bench press, cambered barbell, performance retention, movement velocity, range of motion, exercise-specific warm-up

## Abstract

**Background**: Exercise-specific warm-up may influence fatigue-related performance decline and the restoration of performance between repeated resistance-training sets. This study examined whether a cambered-bar warm-up, compared with a standard-bar warm-up, affected first-repetition velocity and power retention and acceleration–time characteristics. **Methods**: This randomized parallel-group study included twenty resistance-trained men randomly allocated to a cambered-bar condition (CAM; *n* = 10) or a standard-bar condition (ST; *n* = 10). The warm-up comprised four sets at 30%, 50%, 60%, and 70% of one-repetition estimated maximum (1RM). After 10 min, participants completed four sets of five standard-bar repetitions at 75% of 1RM, with 2 min inter-set recovery. First-repetition mean concentric velocity retention was the principal outcome. First-repetition power retention, acceleration index, range of motion (ROM), peak velocity, time to peak velocity, and inter-set velocity rebound were analyzed using linear mixed-effects models. **Results**: During the loading protocol, first-repetition mean concentric velocity and power retention were greater in CAM than ST by 9.82 and 11.65 percentage points, respectively (exact participant-level permutation *p* = 0.044 and *p* = 0.012, respectively). No condition × target-set interactions were observed, indicating overall between-condition differences averaged across L2–L4 rather than different set-to-set trajectories. No condition effect was observed for inter-set velocity rebound. CAM increased warm-up ROM by 6.34 cm compared with ST. No condition effects or condition × stage interactions were observed for warm-up acceleration index, peak velocity, or time to peak velocity. The acceleration index increased across loading sets independently of condition. **Conclusions**: A cambered-bar warm-up increased movement range and was associated with greater retention of first-repetition mean concentric velocity and power during repeated bench press sets. However, no evidence was found for a between-condition difference in immediate inter-set velocity rebound. These findings suggest that the cambered-bar warm-up condition may support the maintenance of first-repetition mechanical performance across repeated sets, although confirmation in larger randomized crossover studies is required.

## 1. Introduction

Repeated resistance exercise requires not only the generation of high mechanical output but also the ability to maintain mechanical performance across consecutive sets. Acute fatigue during resistance exercise is commonly reflected by reductions in repetition velocity and power, while the magnitude of velocity loss has been associated with the mechanical and metabolic demands imposed by the completed work [1,2]. Recovery during the interval between sets is a multifactorial process involving the partial restoration of metabolic and neuromuscular function. Phosphocreatine resynthesis contributes to the rapid restoration of adenosine triphosphate availability following high-intensity muscular activity, whereas the recovery of voluntary force and velocity also depends on the progressive dissipation of neuromuscular fatigue generated during the preceding set [3,4]. Because phosphocreatine restoration and neuromuscular recovery may remain incomplete during relatively short rest intervals, subsequent performance is influenced by the duration of recovery and the magnitude of the preceding exercise stimulus [3,4]. The extent of performance restoration before the next set depends on several factors, including the duration of the recovery interval, external load, individual strength level, and demands of the preceding set [5,6,7,8]. Because the first repetition of a subsequent set is performed before additional within-set fatigue accumulates, its velocity and power may provide practical indicators of the degree to which performance has been restored during the preceding inter-set recovery period [4,5,6,7,8]. However, when these values are expressed relative to the first repetition of L1, they quantify the retention of performance across the loading protocol rather than the amount of performance restored during each individual inter-set interval. The immediate change across a recovery interval can instead be described by comparing the final repetition of the preceding set with the first repetition of the subsequent set.

The ability to preserve performance across repeated sets may also be influenced by the preparation performed before the main exercise. Acute improvements in voluntary performance following a preceding conditioning activity are commonly interpreted within the framework of post-activation performance enhancement (PAPE). Although such responses have historically often been described as post-activation potentiation (PAP), PAP specifically refers to an increase in electrically evoked twitch force following a conditioning contraction, whereas PAPE describes changes in subsequent voluntary performance [9,10]. PAPE may reflect the combined influence of potentiation-related processes, increased muscle temperature, changes in muscle water content, and alterations in neural activation. Whether performance is enhanced depends on the balance between these potentially facilitatory responses and the fatigue generated by the conditioning activity. This balance may be affected by the load and volume of the conditioning activity, the recovery interval, and the strength and training status of the individual [9,10]. Exercise-specific warm-up is widely used before resistance training; however, its effects depend on the applied load, volume, and progression [11]. A progressive bench press warm-up incorporating moderate and high loads reduced the time required to reach peak velocity and increased subsequent mechanical work compared with a low-load warm-up alone [11]. Similarly, a high-load, low-volume warm-up enhanced the training volume completed during a subsequent resistance exercise session [12]. Specific warm-up has also been shown to increase movement velocity during the initial repetitions of subsequent bench press exercise [13]. These findings indicate that warm-up may influence not only initial readiness but also the capacity to maintain mechanical performance during the subsequent training protocol. However, previous research has focused primarily on the load and volume of the warm-up, while the potential role of movement range as a warm-up variable remains less understood.

Manipulating the bench press range of motion (ROM) may provide an additional means of modifying the warm-up stimulus [14]. A cambered barbell allows the bar to descend below the position permitted by a standard straight bar, thereby increasing barbell displacement and exposing the performer to a greater movement range. Bench press performed with a cambered bar has been shown to produce a greater range of motion and longer time under load, accompanied by higher bar velocity and power under selected loading conditions [14]. The use of a cambered bar may also alter the distribution of muscle excitation compared with the standard-bar bench press [15]. More generally, changes in bench press range of motion influence the load–velocity relationship, the contribution of the propulsive and braking phases, and the characteristics of the sticking region [16]. Thus, an increased-range-of-motion warm-up represents a mechanically distinct preparatory stimulus rather than merely a variation in the external load.

The greater displacement associated with the cambered bar may increase the distance available for barbell acceleration and provide preparation across a broader movement range [16]. Within the PAPE framework, the mechanically distinct stimulus produced by the cambered-bar warm-up could theoretically modify the balance between fatigue and subsequent voluntary performance enhancement [9,10]. However, because the present study did not assess neuromuscular, thermal, metabolic, or contractile mechanisms, PAPE should be considered a plausible theoretical framework rather than a mechanism demonstrated by the present data. Conversely, the additional displacement may increase the mechanical demands of the warm-up and potentially influence subsequent performance restoration [14,17,18]. During repeated sets performed to volitional exhaustion, cambered- and standard-bar bench press produced similar reductions in mean velocity and similar post-exercise performance responses, despite the greater range of motion achieved with the cambered bar [17]. However, it remains unknown whether using a cambered bar exclusively during the warm-up affects the restoration and preservation of performance during subsequent repeated sets performed with a standard bar.

Mean velocity and mean power summarize mechanical performance across the concentric phase of an individual repetition, whereas set-averaged values aggregate performance across multiple repetitions. However, these variables do not fully describe the temporal development of velocity within a repetition. Acceleration index, peak velocity, and time to peak velocity may provide complementary information regarding the temporal organization of barbell motion. For example, variable resistance altered barbell acceleration and the velocity–time profile during bench press throws, even when differences in peak and mean power were small or unclear [19]. Assessment of acceleration–time characteristics may therefore identify changes in movement execution that are not apparent in set-averaged performance measures alone. When interpreted together with first-repetition performance, these variables may also contribute to the characterization of fatigue-related changes during repeated resistance exercise and the adequacy of inter-set mechanical retention [19,20].

Although the effects of warm-up load, inter-set rest duration, and bench press range of motion have been investigated separately, little is known about whether an increased-range-of-motion warm-up modifies first-repetition mechanical performance retention during subsequent standard-bar exercise. Therefore, the present study examined the acceleration–time characteristics of progressive bench press warm-ups performed with either a cambered or standard barbell and evaluated first-repetition mean concentric velocity and power retention during four subsequent standard-bar bench press sets performed at 75% of the one-repetition maximum (1RM). Range of motion was assessed during the progressive warm-up as a manipulation check, while acceleration index, peak velocity, and time to peak velocity were used to characterize barbell motion. During the subsequent loading protocol, first-repetition mean concentric velocity retention was considered the principal outcome, with first-repetition power retention treated as a complementary outcome. Inter-set velocity rebound was examined separately as a secondary performance-based indicator of the immediate change occurring across each 2 min recovery interval. It was hypothesized that the cambered-bar warm-up would produce the expected increase in range of motion, modify acceleration–time characteristics during the progressive warm-up, and result in greater retention of first-repetition mean concentric velocity and power during the subsequent repeated-set protocol.

## 2. Materials and Methods

### 2.1. Study Design

This randomized, two-arm, parallel-group study compared two exercise-specific bench press warm-up protocols. Participants completed two laboratory visits separated by 7 days. The first visit included familiarization with the study procedures and the estimated 1RM assessment, whereas the second visit comprised the experimental protocol. Participants performed the progressive warm-up using either a cambered barbell (CAM) or a standard straight barbell (ST). Following a standardized general warm-up, participants completed four progressively loaded bench press warm-up sets at 30%, 50%, 60%, and 70% of 1RM. After a standardized 10 min recovery interval, all participants completed four sets of five bench press repetitions at 75% 1RM using a standard straight barbell, with 2 min recovery intervals between consecutive sets. Mechanical variables were recorded throughout the exercise-specific warm-up and loading protocol using a linear position transducer. The analyses focused on acceleration–time characteristics during the progressive warm-up and first-repetition mechanical performance retention during the subsequent repeated-set protocol. Inter-set velocity rebound was analyzed separately as a secondary performance-based indicator of the immediate change occurring across each recovery interval. Because this was an acute randomized laboratory study evaluating immediate mechanical responses rather than a clinical intervention intended to assess therapeutic, preventive, or rehabilitative effects, the study was not prospectively registered.

### 2.2. Participants

**Figure 1:**
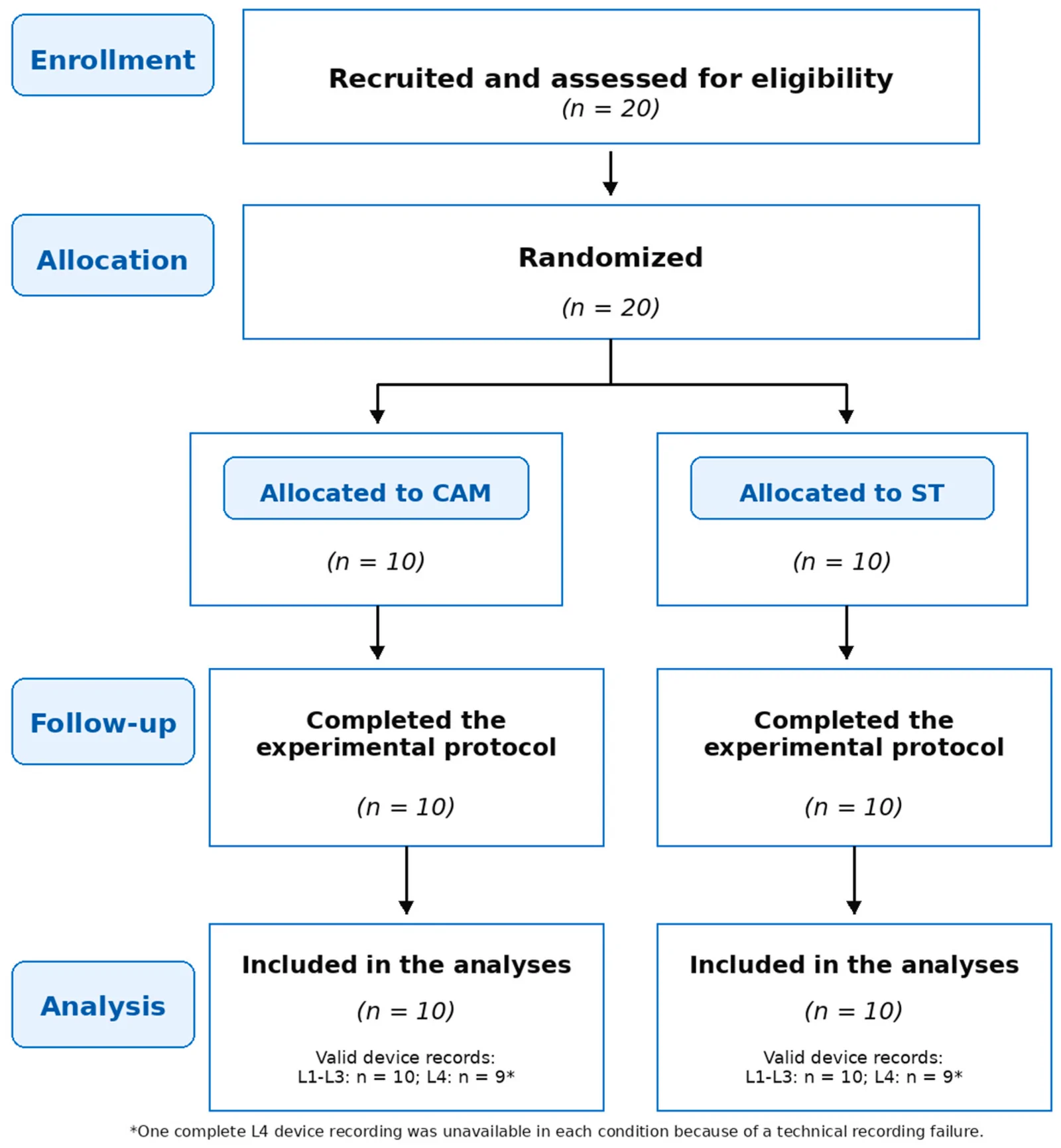
Participant flow diagram.

Twenty resistance-trained men, aged 20–24 years, participated in the study and were randomly allocated to either the CAM condition (*n* = 10) or the ST condition (*n* = 10). Participants were recruited during the month preceding the first laboratory visit. Eligibility criteria included at least two years of consistent resistance-training experience and regular performance of the flat barbell bench press. All participants also reported previous experience performing the bench press with a cambered barbell. Participants were excluded if they reported a current or recent upper-limb musculoskeletal injury, a medical condition contraindicating high-intensity resistance exercise, or the use of medications or substances that could affect physical performance. Before testing, participants were instructed to refrain from upper-body resistance training for 48 h and to avoid alcohol and caffeine for 24 h. Participants were allocated to the CAM or ST condition in a 1:1 ratio using a computer-generated simple randomization sequence. No blocking or stratification was applied. Because this was a single-investigator study, the same investigator generated the allocation sequence, enrolled the participants, and assigned the intervention condition. No independent allocation-concealment procedure was used. Blinding was not feasible during data collection because the barbell type was directly visible, and data processing and statistical analysis were not performed under blinded condition labels. Mechanical variables were recorded automatically using a linear position transducer and processed according to standardized procedures. The a priori sample-size calculation was performed using G*Power, version 3.1.9.2 (Kiel University, Kiel, Germany) [21,22]. The calculation was based on an *F* test for a condition × repeated-measures interaction and assumed two conditions and four repeated measurements. An effect size of *f* = 0.30 was selected as a moderate planning value, with the anticipated magnitude informed by the strength-training-specific effect-size framework proposed by Rhea [23]. A within-participant correlation of 0.80 was assumed to reflect the expected high consistency of repeated bench press velocity and power measurements, consistent with previous test–retest reliability evidence for these mechanical outcomes obtained using a linear position transducer [24]. A nonsphericity correction of ε = 0.90 was applied to allow for a modest departure from perfect sphericity. With an α level of 0.05 and statistical power of 0.80, the minimum required sample was 18 participants. The calculation did not include an additional allowance for missing data. The final sample included 20 participants. The study was conducted in accordance with the Declaration of Helsinki and was approved by the Institutional Review Board of Wroclaw University of Health and Sport Sciences (approval no. 06/2023). All participants were informed about the study aims, procedures, potential risks, and benefits and provided written informed consent before participation. The study sample flow is presented in Figure 1.

### 2.3. Anthropometric Measurements

Body height was measured using a standard anthropometer (GPM Anthropological Instruments, DKSH Ltd., Zürich, Switzerland). Body mass was measured using a bioelectrical impedance analyzer (InBody 230, InBody Co., Ltd., Cerritos, CA, USA) [25,26].

Body mass index was calculated as:

BMI (kg·m^−2^) = body mass (kg)/height^2^ (m^2^)

### 2.4. Bench Press Setup and Mechanical Measurements

All testing was performed under controlled laboratory conditions. Both the cambered barbell and the standard straight barbell had a mass of 20 kg. Barbell mass was included when setting the prescribed external loads, ensuring that the same absolute resistance was applied in both warm-up conditions. The cambered barbell was a Cambered Bar GCB015 GRAND (GRAND, Krzykosy, Poland), with an overall length of 220 cm, a 28 mm shaft diameter, and 50-mm sleeves and a camber depth of 10 cm. The standard straight Olympic barbell was a GRAND (product code A0135-N; GRAND, Krzykosy, Poland), also with an overall length of 220 cm, a 28 mm shaft diameter, and 50 mm sleeves. Thus, both barbells had the same total mass, overall length, shaft diameter, and sleeve diameter, and were compatible with the same Olympic weight plates. In both conditions, the bottom position was defined by controlled contact between the central portion of the barbell and the chest, without bouncing.

Barbell displacement and mechanical variables were recorded using a linear position transducer (Vitruve VBT, SPEED4LIFTS S.L., Madrid, Spain) [27,28,29] attached vertically to the barbell. The device continuously recorded barbell displacement and mean and peak concentric velocity during each repetition. Grip width was standardized at 150% of the participant’s biacromial width and replicated throughout all testing procedures. All repetitions were performed using a standardized 2-0-X-0 tempo, comprising a 2 s eccentric phase, no intentional pause at the bottom position, a concentric phase performed with maximal intended velocity, and no intentional pause after completion of the repetition. Participants maintained stable foot placement and continuous contact with the bench. Bouncing the barbell against the chest was not permitted. Spotters supervised all sets and monitored exercise technique and safety.

### 2.5. Bench Press One-Repetition Maximum Assessment

The estimated 1RM assessment was conducted during the first laboratory visit, which also served as the familiarization session. The experimental protocol was performed during the second visit, 7 days later. The estimated 1RM was generated automatically by the Vitruve software (version 2.4.3; SPEED4LIFTS S.L., Madrid, Spain) from the participant-specific load–velocity relationship established during the incremental test; no external equation, spreadsheet, or manual calculation was used by the investigator. Bench press 1RM was estimated using an incremental load–velocity procedure. Barbell velocity was recorded using the same linear position transducer subsequently used during the experimental protocol [27,28,29]. All relative loads used during the experimental session were prescribed according to the estimated standard-bar bench press 1RM. Participants initially completed 5 min of stationary cycling at a rating of perceived exertion of 4–5. This was followed by 20 alternating arm circles in each direction, three sets of 10 bodyweight push-ups performed at a controlled tempo with 30 s recovery intervals, and two sets of 15 alternating horizontal resistance-band presses performed continuously at a rating of perceived exertion of 2–3. The exercise-specific preparation consisted of 12–15 repetitions using an unloaded barbell, 10–12 repetitions at approximately 40% of the predicted 1RM, and two or three sets of five repetitions at approximately 60–80% of the predicted 1RM. The incremental test commenced at approximately 80% of the predicted 1RM. The external load was increased by 5–10% between successive attempts. Three repetitions were initially performed. The number of repetitions was reduced to two when mean concentric velocity decreased below approximately 0.50 m·s^−1^ and to one when it decreased below approximately 0.25 m·s^−1^. The test was terminated after a maximum of five sets or when the participant could no longer complete a repetition using the prescribed technique. Recovery intervals of 3–5 min were provided between attempts. All repetitions were performed using the standardized grip width, controlled eccentric phase, maximal concentric intent, and without barbell bouncing. Participants received real-time velocity feedback during the test. Relative bench press strength was calculated as: relative strength = bench press 1RM (kg)/body mass (kg).

### 2.6. General Warm-Up

The experimental session began with 5 min of stationary cycling at a rating of perceived exertion of 4–5. Participants then performed 20 alternating arm circles in each direction, three sets of 10 bodyweight push-ups at a controlled tempo with 30 s recovery intervals, and two sets of 15 alternating horizontal resistance-band presses performed continuously at a rating of perceived exertion of 2–3. A 3 min transition period was provided before the exercise-specific bench press warm-up.

### 2.7. Exercise-Specific Warm-Up

Participants performed the progressive bench press warm-up according to their allocated condition. The CAM group performed all exercise-specific warm-up sets using a cambered barbell, whereas the ST group used a standard straight barbell.

The progressive warm-up comprised:

W1: 12 repetitions at 30% 1RM;

W2: 10 repetitions at 50% 1RM;

W3: 8 repetitions at 60% 1RM;

W4: 5 repetitions at 70% 1RM.

A 2 min recovery interval was provided between consecutive warm-up sets. All repetitions were performed using the standardized grip width, 2-0-X-0 movement tempo, and maximal intended concentric velocity.

Because the external load increased while the prescribed number of repetitions decreased from W1 to W4, the warm-up stages represented different combinations of load and volume. Changes across W1–W4 were, therefore, interpreted as responses to the complete progressive warm-up sequence rather than as isolated effects of external load, set volume, or fatigue.

### 2.8. Loading Protocol

Following a standardized 10 min recovery interval after the exercise-specific warm-up, all participants completed four sets of five bench press repetitions at 75% estimated 1RM using a standard straight barbell. Recovery intervals of 2 min were provided between sets. The grip width, movement tempo, technical requirements, and instruction to perform the concentric phase with maximal intended concentric velocity were maintained throughout the loading protocol.

### 2.9. Mechanical Variables

Mean concentric velocity represented the mean barbell velocity recorded during the concentric phase of each repetition and was expressed in meters per second. First-repetition mean concentric velocity was used to calculate velocity retention, while the mean concentric velocities of the final and first repetitions of consecutive sets were used to calculate inter-set velocity rebound.

Peak velocity was defined as the highest instantaneous barbell velocity recorded during the concentric phase and was expressed in meters per second.

Time to peak velocity represented the elapsed time from the beginning of the concentric phase until peak barbell velocity was reached and was expressed in milliseconds.

The acceleration index was obtained directly from the device export and was expressed in meters per second squared.

Mean power represented the mean power output recorded during the concentric phase of each repetition and was expressed in watts. First-repetition mean power was used to calculate power retention during L2–L4.

Barbell range of motion represented the vertical displacement of the barbell during the recorded movement and was expressed in centimeters. During the progressive warm-up, range of motion was treated as a manipulation-check variable used to verify that the cambered bar produced the intended increase in barbell displacement.

### 2.10. Data Processing and Outcome Classification

The progressive warm-up and loading phases were analyzed separately. The warm-up stages differed in both external load and prescribed repetition number, whereas the four loading sets were performed using the same load and repetition structure. For acceleration index, range of motion, peak velocity, and time to peak velocity, the set-level value represented the arithmetic mean of the repetition-level values recorded within the respective set. Stage-level averaging was used because the aim was to characterize the overall mechanical response to each complete stage of the prescribed progressive warm-up rather than repetition-to-repetition changes within a set. The stages intentionally differed in relative load and prescribed repetition number and were, therefore, treated as distinct components of the warm-up protocol. The acceleration index was analyzed as an absolute set-level outcome across W1–W4 and L1–L4.

Changes in peak velocity and time to peak velocity during the progressive warm-up were calculated relative to W1:

ΔW2 = W2 − W1;

ΔW3 = W3 − W1;

ΔW4 = W4 − W1.

Corresponding changes during the loading protocol were calculated relative to L1:

ΔL2 = L2 − L1;

ΔL3 = L3 − L1;

ΔL4 = L4 − L1.

Positive values indicated that the outcome was higher during the target stage or set than during the corresponding reference set. Negative values indicated a reduction relative to the reference set.

### 2.11. First-Repetition Performance Retention

Mechanical performance retention across the loading protocol was assessed using the first repetition of each loading set. The first repetition was selected because it was performed immediately after the prescribed recovery interval and before additional within-set performance decline could accumulate. These outcomes quantified how closely first-repetition performance during L2–L4 remained to the value recorded during L1; they did not quantify the amount of performance restored during each individual recovery interval.

First-repetition mean concentric velocity retention during L2, L3, and L4 was calculated relative to the first repetition of L1:

First-repetition mean concentric velocity retention (%) = [MCV(target set, repetition 1)/MCV(L1, repetition 1)] × 100

A value of 100% indicated that first-repetition mean concentric velocity in the target set was equal to that recorded in L1. Values below 100% indicated lower first-repetition performance relative to L1, whereas values above 100% indicated that first-repetition mean concentric velocity exceeded the L1 value.

First-repetition power retention was calculated using the same procedure:

First-repetition power retention (%) = [MP(target set, repetition 1)/MP(L1, repetition 1)] × 100

In this equation, MP represents mean concentric power recorded during the respective repetition.

First-repetition mean concentric velocity retention was considered the principal outcome of mechanical performance retention. First-repetition power retention was treated as a complementary outcome.

### 2.12. Inter-Set Velocity Rebound

Inter-set velocity rebound was examined as a secondary performance-based indicator of the immediate change occurring across the 2 min recovery interval. It was calculated as the difference between the first repetition of the subsequent set and the final repetition of the preceding set:

Velocity rebound (m·s^−1^) = MCV(next set, repetition 1) − MCV(previous set, final repetition)

Velocity rebound was calculated for the L1 → L2, L2 → L3, and L3 → L4 transitions. Positive values indicated that barbell velocity increased during the recovery interval. Negative values indicated that the velocity of the first repetition of the subsequent set remained below the final-repetition velocity of the preceding set. Inter-set velocity rebound was treated as a secondary outcome because its calculation required both the final repetition of the preceding set and the first repetition of the subsequent set to be available.

### 2.13. Statistical Analysis

Participant characteristics were summarized separately for the CAM and ST groups using means, standard deviations, and 95% confidence intervals. Because group allocation was randomized, no inferential comparisons of baseline participant characteristics were performed. Study outcomes were summarized separately for each condition and measurement stage as means, standard deviations, and 95% confidence intervals. All data processing and inferential analyses were performed using Python, version 3.13.5, with pandas, version 2.2.3, NumPy, version 2.3.5, statsmodels, version 0.14.6, and SciPy, version 1.17.0. Statistical significance was set at *p* < 0.05; all tests were two-sided.

Separate linear mixed-effects models were fitted for each dependent variable using restricted maximum likelihood estimation. The participant was included as a random intercept to account for repeated observations within individuals. Each model included the experimental condition, the relevant categorical repeated factor, and their interaction as fixed effects:

Outcome ~ condition × repeated factor + (1 | participant)

Condition and repeated-measures factors were entered as categorical variables using treatment coding. ST was used as the reference condition. The first analyzed level of the repeated factor was used as the reference category: W1 or L1 for absolute outcomes and W2 or L2 for change and retention outcomes. The models were fitted using the BFGS optimizer and the default statsmodels convergence criteria. All reported models converged successfully; no singular fits were identified.

For acceleration index, the repeated factor comprised W1–W4 during the progressive warm-up and L1–L4 during the loading protocol. Warm-up range of motion was analyzed using the same model structure as a manipulation check. The range-of-motion results were not interpreted as primary evidence of warm-up efficacy. Changes in peak velocity and time to peak velocity were analyzed across W2–W4 or L2–L4, with all values expressed relative to W1 or L1, respectively. First-repetition mean concentric velocity and power retention were analyzed across L2–L4. Inter-set velocity rebound was analyzed across the L1 → L2, L2 → L3, and L3 → L4 transitions. Fixed effects were evaluated using approximate Wald *F* tests. The numerator degrees of freedom corresponded to the rank of the tested fixed-effect term, whereas the denominator degrees of freedom were calculated as the number of valid observations minus the number of estimated fixed-effect coefficients. For example, the retention and loading change models included 58 valid observations and six fixed-effect coefficients, resulting in 52 denominator degrees of freedom. The original Wald tests did not incorporate Satterthwaite or Kenward–Roger small-sample corrections. These residual degrees of freedom do not represent participant-level degrees of freedom for the between-participant condition contrast and do not provide a cluster-based small-sample correction for that effect. F statistics are reported with numerator and denominator degrees of freedom for the mixed-model effects. When a statistically significant omnibus effect was identified, pairwise comparisons of model-estimated marginal means were performed. The Holm procedure was used to adjust *p* values within each family of comparisons. Model results are presented as F values, *p* values, estimated marginal means or contrasts, and 95% confidence intervals. To complement the model-estimated contrasts expressed in the original measurement units, standardized between-condition effect sizes were calculated as Hedges’ *g*. Model fit was summarized using marginal and conditional coefficients of determination. Marginal and conditional R^2^ were calculated using a Nakagawa-style variance decomposition. Marginal R^2^ represented the variance attributable to the fixed effects divided by the total variance, whereas conditional R^2^ represented the variance attributable to the fixed and random effects combined divided by the total variance. The intraclass correlation coefficient was calculated from the participant-level random-intercept variance and residual variance. Model assumptions were assessed primarily by visual inspection of residual-versus-fitted plots and Q–Q plots. The residual-versus-fitted plots did not indicate substantial systematic patterns; the Q–Q plots did not reveal major departures from the expected residual distribution in the principal models. Residual skewness, excess kurtosis, and the Shapiro–Wilk test were used only as supplementary diagnostic measures. Because the residual degrees of freedom used in the Wald tests did not provide participant-level small-sample inference for the between-participant condition effect, exact participant-level permutation tests were treated as the primary inferential procedure for the three between-condition effects supporting the principal findings. Exact permutation tests were performed across all 184,756 possible allocations of 20 participants into two groups of ten. The permutation statistic was based on the between-condition difference in participant-level mean values across the available L2–L4 measurements. Participant-level percentile bootstrap confidence intervals were also calculated using 100,000 stratified resamples within condition. The mixed-effects models were retained as the primary framework for estimating model-adjusted condition differences and for evaluating repeated-factor and interaction effects, whereas the permutation tests provided the primary small-sample inference for the reported between-condition findings. Missing observations were not imputed. The linear mixed-effects models used all available valid observations, enabling participants with an unavailable measurement at one stage to contribute their remaining observations. All 20 participants were retained in the analyses.

## 3. Results

### 3.1. Data Completeness and Quality Control

All 20 participants completed all prescribed sets and repetitions. Complete device recordings were available for all participants during the progressive warm-up, corresponding to 120, 100, 80, and 50 recorded repetitions per condition at W1, W2, W3, and W4, respectively. During the loading protocol, valid recordings were available for 10 participants per condition at L1–L3, corresponding to 50 repetitions per condition at each set. At L4, the complete linear position transducer record was unavailable for one participant in each condition because of a technical recording failure; therefore, valid L4 data comprised nine participants and 45 repetitions per condition. The missing records were unrelated to fatigue, exercise execution, or an inability to complete the set. The same data availability applied to all device-derived outcomes assessed at the respective stages.

### 3.2. Participant Characteristics

Participant characteristics are presented in Table 1. No inferential comparisons of baseline characteristics were performed because participants were randomly allocated to the experimental conditions.

### 3.3. Progressive Warm-Up

Descriptive results for the progressive warm-up are presented in Table 2. Mixed-model results for the acceleration–time outcomes are summarized in Table 3.

### 3.4. Range-of-Motion Manipulation Check

The cambered-bar warm-up produced the intended increase in barbell displacement. A significant condition effect was observed for range of motion, F = 9.09, *p* = 0.004. Averaged across the four warm-up stages, the model-estimated range of motion was 49.68 cm in CAM and 43.34 cm in ST, corresponding to an adjusted CAM–ST difference of 6.34 cm (95% CI [2.15, 10.53]). There was also a warm-up-stage effect, F = 17.49, *p* < 0.001, whereas the condition × stage interaction was not significant, F = 0.59, *p* = 0.624. Averaged across conditions, range of motion decreased from 49.24 cm at W1 to 46.68 cm at W2, 46.09 cm at W3, and 44.03 cm at W4. The condition effect, therefore, confirmed the intended experimental manipulation.

### 3.5. Acceleration Index—Warm Up

There was no main effect of condition on the acceleration index, F = 1.00, *p* = 0.321, and no condition × warm-up-stage interaction, F = 0.32, *p* = 0.811. A significant warm-up-stage effect was observed, F = 6.12, *p* < 0.001. Averaged across conditions, the model-estimated acceleration index was 6.09 m·s^−2^ at W1, 3.47 m·s^−2^ at W2 3.30 m·s^−2^ at W3, and 3.90 m·s^−2^ at W4. The acceleration index was lower at W2 than at W1 (adjusted difference = −2.62 m·s^−2^, 95% CI [−4.10, −1.15], Holm-adjusted *p* = 0.003), lower at W3 than at W1 (adjusted difference = −2.79 m·s^−2^, 95% CI [−4.26, −1.31], *p* = 0.002), and lower at W4 than at W1 (adjusted difference = −2.19 m·s^−2^, 95% CI [−3.67, −0.72], *p* = 0.016).

### 3.6. Time to Peak Velocity—Warm Up

Changes in time to peak velocity relative to W1 did not differ between CAM and ST, F < 0.01, *p* = 0.996. The condition × target-stage interaction was also not significant, F = 2.17, *p* = 0.124. A significant target-stage effect was observed, F = 9.67, *p* < 0.001. Averaged across conditions, time to peak velocity increased by 140.53 ms at W2, 212.71 ms at W3, and 325.52 ms at W4, relative to W1. The increase at W4 was greater than at W2 (adjusted difference = 185.00 ms, 95% CI [99.96, 270.03], Holm-adjusted *p* < 0.001) and W3 (adjusted difference = 112.81 ms, 95% CI [27.78, 197.84], *p* = 0.021).

### 3.7. Peak Velocity—Warm Up

Changes in peak velocity relative to W1 showed no condition effect, F = 1.31, *p* = 0.258, and no condition × target-stage interaction, F < 0.01, *p* = 0.999. A significant target-stage effect was observed, F = 75.64, *p* < 0.001. The model-estimated change in peak velocity became progressively more negative, from −0.536 m·s^−1^ at W2 to −0.784 m·s^−1^ at W3 and −0.966 m·s^−1^ at W4. All pairwise comparisons were statistically significant after Holm adjustment: W3 versus W2, adjusted difference = −0.248 m·s^−1^, *p* < 0.001; W4 versus W2, adjusted difference = −0.431 m·s^−1^, *p* < 0.001; and W4 versus W3, adjusted difference = −0.182 m·s^−1^, *p* < 0.001.

### 3.8. Loading Protocol Outcomes Analysis

Descriptive results for the loading protocol are presented in Table 4 and supplemented by the absolute first-repetition mean concentric velocity and mean power values reported below, while the mixed-model results are summarized in Table 5.

### 3.9. Acceleration Index—Loading Protocol

There was no condition effect for acceleration index, F < 0.01, *p* = 0.985, and no condition × loading-set interaction, F = 0.71, *p* = 0.548. A significant loading-set effect was observed, F = 3.23, *p* = 0.027. Averaged across conditions, the model-estimated acceleration index was 4.95 m·s^−2^ at L1, 6.70 m·s^−2^ at L2, 7.37 m·s^−2^ at L3, and 8.30 m·s^−2^ at L4. The acceleration index was greater at L4 than at L1 (adjusted difference = 3.35 m·s^−2^, 95% CI [1.11, 5.59], Holm-adjusted *p* = 0.024). No other pairwise comparison remained statistically significant after Holm adjustment.

### 3.10. Absolute First-Repetition Mean Concentric Velocity and Mean Power—Loading Protocol

Absolute first-repetition mean concentric velocity and mean power values are reported descriptively to contextualize the retention outcomes calculated relative to L1. In CAM, first-repetition mean concentric velocity was 0.44 ± 0.18 m·s^−1^ (95% CI [0.31, 0.57]) at L1, 0.45 ± 0.17 m·s^−1^ (95% CI [0.33, 0.57]) at L2, 0.40 ± 0.15 m·s^−1^ (95% CI [0.30, 0.51]) at L3, and 0.45 ± 0.19 m·s^−1^ (95% CI [0.30, 0.59]) at L4. The corresponding values in ST were 0.42 ± 0.09 m·s^−1^ (95% CI [0.35, 0.48]), 0.39 ± 0.07 m·s^−1^ (95% CI [0.34, 0.44]), 0.37 ± 0.08 m·s^−1^ (95% CI [0.31, 0.43]), and 0.36 ± 0.09 m·s^−1^ (95% CI [0.29, 0.43]), respectively.

First-repetition mean power in CAM was 319.1 ± 146.7 W (95% CI [214.1, 424.0]) at L1, 330.7 ± 145.5 W (95% CI [226.6, 434.8]) at L2, 304.9 ± 142.2 W (95% CI [203.2, 406.6]) at L3, and 327.9 ± 163.3 W (95% CI [202.4, 453.5]) at L4. The corresponding values in ST were 275.6 ± 88.9 W (95% CI [212.0, 339.2]), 252.2 ± 71.1 W (95% CI [201.4, 303.1]), 247.1 ± 73.9 W (95% CI [194.3, 300.0]), and 231.2 ± 80.1 W (95% CI [169.7, 292.8]), respectively. L4 descriptive values were based on nine participants in each condition.

### 3.11. First-Repetition Mean Concentric Velocity Retention—Loading Protocol

**Figure 2:**
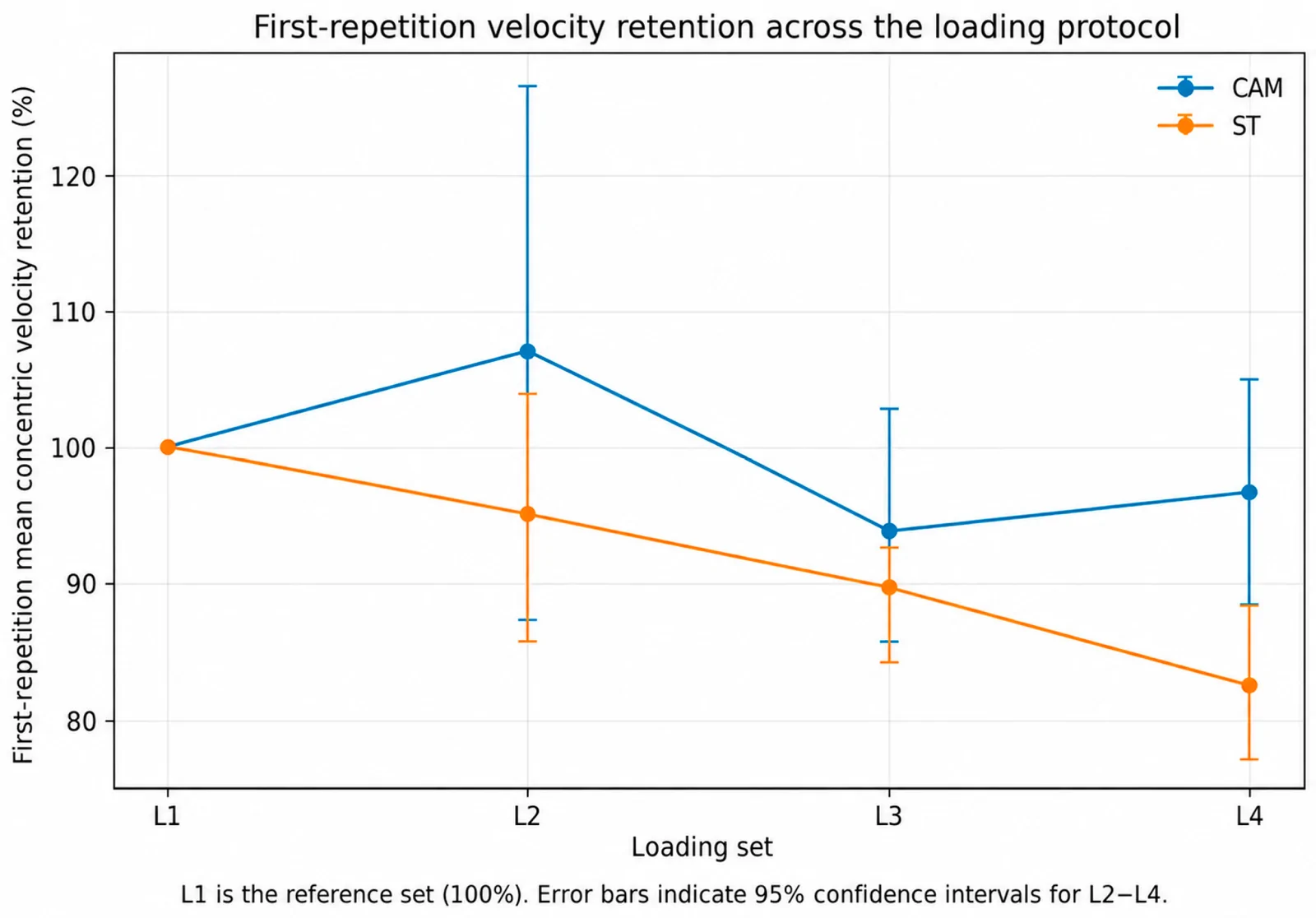
First-repetition mean concentric velocity retention across loading sets L1–L4 in the cambered-bar (CAM) and standard-bar (ST) warm-up conditions. Values are presented as means with 95% confidence intervals. L1 was set as the reference value (100%).

The exact participant-level permutation test supported a between-condition difference in first-repetition mean concentric velocity retention (*p* = 0.044). The corresponding mixed-model Wald test was F (1, 52) = 4.56, *p* = 0.038, as reported in Table 5. Averaged across L2–L4, the model-estimated retention was 99.03% in CAM and 89.21% in ST. The adjusted CAM–ST difference was 9.82 percentage points (95% CI [0.59, 19.04]) with a standardized between-condition effect of Hedges’ *g* = 0.88. The corresponding participant-level stratified bootstrap 95% confidence interval for the CAM–ST difference was [1.49, 18.33] percentage points. A significant target-set effect was also observed, F = 4.50, *p* = 0.016, whereas the condition × target-set interaction was not significant, F = 0.73, *p* = 0.486. Averaged across conditions, model-estimated retention was 101.07% at L2, 91.82% at L3, and 89.47% at L4. Retention was lower at L4 than at L2 (adjusted difference = −11.60 percentage points, 95% CI [−19.93, −3.27], Holm-adjusted *p* = 0.022). Because the interaction was not significant, the condition effect represents an overall difference in first-repetition mean concentric velocity retention averaged across L2–L4 rather than a different pattern of change across successive sets. First-repetition mean concentric velocity retention across L1–L4 in the CAM and ST conditions is presented in Figure 2.

### 3.12. First-Repetition Power Retention—Loading Protocol

The exact participant-level permutation test indicated a between-condition difference in first-repetition power retention (*p* = 0.012). The corresponding mixed-model Wald test was F (1, 52) = 6.94, *p* = 0.011. Averaged across L2–L4, model-estimated retention was 100.26% in CAM and 88.61% in ST, corresponding to an adjusted CAM–ST difference of 11.65 percentage points (95% CI [2.77, 20.53]) and a standardized between-condition effect of Hedges’ *g* = 1.09. The corresponding participant-level stratified bootstrap 95% confidence interval for the CAM–ST difference was [3.55, 19.86] percentage points. A significant target-set effect was also observed, F = 4.25, *p* = 0.020, whereas the condition × target-set interaction was not significant, F = 0.73, *p* = 0.486. Averaged across conditions, model-estimated retention was 100.90% at L2, 93.36% at L3, and 89.04% at L4. Retention was lower at L4 than at L2 (adjusted difference = −11.86 percentage points, 95% CI [−20.18, −3.54], Holm-adjusted *p* = 0.018). As with velocity retention, the condition effect represents an overall difference averaged across L2–L4 and not a statistically different set-to-set trajectory between CAM and ST.

### 3.13. Time to Peak Velocity—Loading Protocol

The exact participant-level permutation test indicated a between-condition difference in the change in time to peak velocity (*p* = 0.045). The corresponding mixed-model Wald test was F (1, 52) = 4.23, *p* = 0.045. Averaged across L2–L4, the model-estimated change was −4.35 ms in CAM and −198.29 ms in ST, corresponding to an adjusted CAM–ST difference of 193.94 ms (95% CI [4.79, 383.08]) and a standardized between-condition effect of Hedges’ *g* = 0.92. The corresponding participant-level stratified bootstrap 95% confidence interval for the CAM–ST difference was [33.47, 376.58] ms. There was no target-set effect, F = 0.37, *p* = 0.694, and no condition × target-set interaction, F = 0.19, *p* = 0.831.

### 3.14. Peak Velocity and Inter-Set Velocity Rebound—Loading Protocol

No statistically significant condition, repeated-factor, or interaction effects were observed for changes in peak velocity or inter-set velocity rebound. Therefore, the warm-up condition did not significantly influence the change in peak velocity relative to L1 or the immediate increase in velocity from the final repetition of one set to the first repetition of the subsequent set.

### 3.15. Participant-Level Small-Sample Inference

Exact participant-level permutation tests were used as the primary small-sample inference for the three observed between-condition effects, whereas participant-level stratified bootstrap confidence intervals were used to quantify the uncertainty of the corresponding CAM–ST differences. The outcome-specific permutation *p* values and bootstrap confidence intervals are reported alongside the respective mixed-model results in Section 3.11, Section 3.12 and Section 3.13. The velocity-retention and time-to-peak-velocity findings remained close to the conventional significance threshold and should, therefore, be interpreted cautiously.

## 4. Discussion

The present study examined whether a progressive warm-up performed with a cambered barbell influenced first-repetition mechanical performance retention during repeated standard-bar bench press exercise. The main finding was that first-repetition mean concentric velocity and power retention across L2–L4 were 9.82 and 11.65 percentage points higher, respectively, in CAM than in ST. However, the condition effect for mean concentric velocity retention should be interpreted cautiously because its 95% confidence interval was wide and the lower bound was close to zero (0.59 percentage points). In addition, the fixed effects explained a relatively modest proportion of the variance in this outcome (R^2^m = 0.203), indicating substantial uncertainty regarding the magnitude and robustness of the estimate. The absence of condition × target-set interactions indicates that the warm-up conditions did not produce statistically different set-to-set patterns of performance retention. No condition effect was observed for inter-set velocity rebound; the findings, therefore, do not indicate that CAM produced a greater immediate increase in velocity across the individual recovery intervals. Instead, CAM was associated with greater overall first-repetition performance retention when averaged across L2–L4. The study hypotheses were, therefore, partially supported.

The approximately 6.3 cm greater range of motion during CAM confirmed that the warm-up conditions differed mechanically. Similar increases in barbell displacement have previously been reported during cambered-bar bench press, together with changes in velocity, power output, time under load, and muscle excitation [14,15,17]. Previous research suggests that changes in bench press range of motion may influence the eccentric-to-concentric transition, propulsive phase, sticking region, and contribution of the shoulder and elbow extensors [16,30,31]. However, these findings provide contextual information regarding the mechanical characteristics of full cambered-bar exercise and do not establish the mechanism through which a cambered-bar warm-up may affect subsequent standard-bar performance. In the present study, the directly supported finding was that CAM increased barbell displacement during the warm-up without producing condition-specific differences in the analyzed warm-up acceleration–time outcomes. Thus, the cambered bar provided a mechanically distinct preparatory condition without an evident condition-specific reduction in the analyzed warm-up outcomes.

Changes observed across the progressive warm-up should not be interpreted exclusively as fatigue [31,32]. Peak velocity decreased and time to peak velocity increased, while the external load progressed from 30% to 70% 1RM and the prescribed number of repetitions decreased from 12 to 5 [33]. These responses were, therefore, likely influenced by the inverse load–velocity relationship and the changing combination of load and volume [16,34]. Accordingly, the stage effects cannot be attributed independently to external load, set volume, or fatigue-related performance changes. Moreover, no condition-specific differences in these responses were detected.

The retention outcomes were designed to assess how closely first-repetition performance during L2–L4 remained to the value recorded during L1. Accordingly, they provide indicators of mechanical performance retention across the loading protocol rather than direct measures of the amount of performance restored during each inter-set interval. They also do not directly measure physiological, metabolic, or tissue-level recovery [35]. The higher retention in CAM suggests that the complete cambered-bar warm-up condition may have supported the subsequent maintenance of initial-repetition performance. However, because velocity and power were measured under the same external load, these findings should be considered as closely related expressions of the same mechanical response rather than as independent effects [36].

When averaged across conditions, first-repetition mean concentric velocity and power retention were lower at L4 than at L2. This finding indicates that first-repetition performance during the latter part of the protocol was further below the initial L1 value and is consistent with a fatigue-related reduction in mechanical performance during repeated sets [37]. However, the retention outcomes do not establish that less performance was restored specifically during the recovery interval preceding L4. Previous studies have shown that longer inter-set intervals generally improve velocity maintenance and repetition performance, particularly when repeated sets are performed at moderate-to-high relative loads [5,6,7,8]. Nevertheless, the time required to restore movement velocity varies considerably between individuals and may depend on the preceding set, strength level, training status, and individual capacity to maintain performance [4,5,6,7,8]. Consequently, a 2 min interval may provide sufficient recovery for some individuals while remaining inadequate for others.

The present findings also indicate that performance retention relative to L1 should be distinguished from the immediate change in velocity occurring across an individual recovery interval. No statistically significant condition effect was observed for inter-set velocity rebound. Thus, this outcome provided no evidence that CAM altered the immediate increase in velocity from the final repetition of one set to the first repetition of the next. When averaged across L2–L4, first-repetition performance remained closer to the original L1 value in CAM than in ST. Therefore, the cambered-bar warm-up was associated with the overall retention of performance across the session rather than a statistically detectable difference in immediate inter-set performance recovery.

Several mechanisms may hypothetically explain the higher performance retention following CAM. Exposure to a greater movement range may have prepared participants for force production from a deeper barbell position, modified the eccentric-to-concentric transition, or increased the available distance for acceleration [14,15,16,17,30,31]. Subsequent standard-bar repetitions were then performed over a shorter trajectory than that used during the CAM warm-up, potentially reducing the relative mechanical demands of the loading exercise. These explanations are derived primarily from studies examining exercise performed entirely with a cambered bar and cannot be assumed to represent the mechanism of a cambered-bar warm-up followed by standard-bar loading. Nevertheless, these explanations remain speculative because no electromyographic, kinetic or perceptual measures were collected. They should, therefore, be regarded as hypotheses requiring direct examination in future research. Moreover, because mechanical work, time under tension, and exercise-specific relative intensity were not quantified, the observed findings cannot be attributed exclusively to the greater range of motion. The findings, therefore, demonstrate an association between the warm-up condition and mechanical performance retention but do not establish the mechanisms responsible for this effect. An isolated condition effect was also observed for the change in time to peak velocity. Time to peak velocity remained close to the L1 value in CAM, whereas a greater negative change was estimated in ST. However, the confidence interval for the between-condition contrast was wide, and neither the target-set effect nor the condition × target-set interaction was statistically significant. This result should, therefore, be interpreted cautiously and should not be considered evidence of superior recovery in either condition. The remaining acceleration–time outcomes provide limited additional evidence regarding fatigue and recovery. The acceleration index increased across the loading sets despite simultaneous reductions in velocity and power retention. This divergence suggests that the acceleration index and first-repetition retention reflected different aspects of barbell motion. Consequently, the increase in acceleration index should not be interpreted as improved performance or a more complete inter-set recovery.

From a practical perspective, a cambered bar may be incorporated into a progressive bench press warm-up to increase movement range, with no condition-specific differences detected in the analyzed warm-up acceleration–time outcomes. In the present protocol, its use was followed by greater retention of first-repetition mean concentric velocity and power during four standard-bar sets performed at 75% 1RM with 2 min recovery intervals. Monitoring first-repetition mean concentric velocity may help practitioners to track the retention of mechanical performance relative to an initial reference set across repeated sets and identify when performance is no longer being maintained. However, the findings do not demonstrate faster or more complete recovery during the individual inter-set intervals, improved total-set performance, reduced within-set velocity loss, faster physiological recovery, or reduced injury risk. Cambered-bar warm-ups should be introduced progressively and used primarily in resistance-trained individuals familiar with the greater barbell descent and movement range.

Several limitations should be acknowledged. The small sample included only resistance-trained men, limiting the precision and generalizability of the findings. Although the final sample exceeded the minimum indicated by the a priori calculation, the small number of participants in each condition may have provided insufficient power to detect a smaller condition × target-set interaction. Therefore, the nonsignificant interactions should not be interpreted as evidence that the set-to-set trajectories were equivalent between CAM and ST. The parallel-group design did not fully control for individual differences in strength, technique, mobility, performance-maintenance capacity, and recovery capacity. The percentage-based velocity and power retention outcomes showed greater variability in CAM than in ST, and condition-specific residual variances were not modelled. This residual heteroscedasticity may have affected the precision of the model-based condition-effect estimates. However, participant-level bootstrap resampling was stratified within condition, thereby reducing the influence of unequal between-condition variability on the reported confidence intervals. First-repetition mean concentric velocity and power retention represented relative measures of mechanical performance retention, whereas no physiological, metabolic, perceptual, electromyographic, or kinematic measures were collected. Thus, the study cannot determine whether the observed effects reflected changes in physiological recovery, movement strategy, or other mechanisms. Moreover, the warm-up stages differed simultaneously in relative load and repetition number, preventing separate evaluation of the effects of load, volume, fatigue, and potentiation. One complete L4 recording was unavailable in each condition because of technical device failure, reducing the precision of the final-set estimates. In addition, the findings are specific to four sets of five repetitions performed at 75% 1RM with fixed 2 min recovery intervals and may not generalize to other loading schemes, rest durations, or exercises. The findings should, therefore, be interpreted within the specific protocol examined and confirmed in larger randomized crossover studies incorporating direct measures of fatigue and recovery.

## 5. Conclusions

A progressive warm-up performed with a cambered bar was associated with greater retention of first-repetition mean concentric velocity and mean power during repeated bench press exercise. The increased range of motion during the cambered-bar warm-up was not accompanied by condition-specific differences in the analyzed warm-up acceleration–time characteristics. When averaged across conditions, first-repetition mean concentric velocity and power retention were lower at L4 than at L2, indicating a greater reduction relative to the initial L1 performance level during the latter part of the protocol.

These preliminary findings suggest that the complete cambered-bar warm-up condition may support the overall maintenance of first-repetition performance across subsequent sets. However, no statistically significant condition effect was observed for inter-set velocity rebound; the findings, therefore, do not demonstrate a between-condition difference in immediate performance recovery across the individual inter-set intervals or statistically different set-to-set trajectories. First-repetition mean concentric velocity monitoring may provide a practical method for tracking mechanical performance retention across repeated sets. Larger randomized crossover studies incorporating direct physiological and biomechanical measures are required to confirm these findings and clarify the mechanisms involved.

## Figures and Tables

**Table 1:** Participant characteristics by experimental condition.

Variable	CAM (*n* = 10)	ST (*n* = 10)
Body height (m)	1.81 ± 0.04 [1.78, 1.83]	1.81 ± 0.05 [1.77, 1.85]
Body mass (kg)	81.10 ± 7.31 [75.87, 86.33]	76.60 ± 6.83 [71.71, 81.49]
BMI (kg·m^−2^)	24.90 ± 2.22 [23.31, 26.49]	23.42 ± 2.40 [21.71, 25.14]
Training experience (years)	4.10 ± 1.60 [2.96, 5.24]	3.60 ± 1.17 [2.76, 4.44]
Training sessions per week (n)	3.00 ± 0.94 [2.33, 3.67]	3.00 ± 0.94 [2.33, 3.67]
Session duration (min)	82.00 ± 28.11 [61.89, 102.11]	75.00 ± 14.14 [64.88, 85.12]
Weekly training volume (min·week^−1^)	244.50 ± 114.03 [162.93, 326.07]	231.00 ± 96.75 [161.79, 300.21]
Estimated bench press 1RM (kg)	99.00 ± 18.04 [86.10, 111.90]	88.45 ± 16.70 [76.50, 100.40]
Relative strength (1RM/body mass)	1.22 ± 0.17 [1.10, 1.34]	1.15 ± 0.16 [1.03, 1.27]
Loading-protocol load, 75% 1RM (kg)	74.25 ± 13.53 [64.57, 83.93]	66.36 ± 12.52 [57.40, 75.32]

Data are presented as mean ± SD [95% confidence interval for the mean].

**Table 2:** Descriptive results for the progressive warm-up.

Outcome	Condition	W1	W2	W3	W4
Acceleration index (ms^−2^)	CAM	5.85 ± 2.00 [4.42, 7.28]	2.80 ± 1.44 [1.77, 3.83]	3.04 ± 2.99 [0.91, 5.18]	3.07 ± 2.57 [1.23, 4.91]
	ST	6.33 ± 1.04 [5.59, 7.07]	4.13 ± 1.86 [2.80, 5.46]	3.57 ± 3.12 [1.34, 5.79]	4.73 ± 6.06 [0.39, 9.06]
ROM (cm)	CAM	52.78 ± 3.55 [50.23, 55.32]	49.75 ± 5.17 [46.06, 53.45]	49.49 ± 3.76 [46.80, 52.19]	46.69 ± 4.27 [43.64, 49.75]
	ST	45.69 ± 5.10 [42.04, 49.35]	43.61 ± 6.92 [38.66, 48.56]	42.68 ± 6.15 [38.28, 47.08]	41.37 ± 4.99 [37.80, 44.94]
Δ time to peak velocity (ms)	CAM	Reference	185.4 ± 138.4 [86.4, 284.4]	212.0 ± 241.9 [39.0, 385.1]	282.0 ± 305.4 [63.5, 500.5]
	ST	Reference	95.7 ± 94.8 [27.9, 163.5]	213.4 ± 201.8 [69.1, 357.8]	369.1 ± 278.9 [169.6, 568.6]
Δ peak velocity (ms^−1^)	CAM	Reference	−0.569 ± 0.138 [−0.667, −0.471]	−0.818 ± 0.158 [−0.931, −0.705]	−1.000 ± 0.188 [−1.135, −0.866]
	ST	Reference	−0.502 ± 0.148 [−0.608, −0.397]	−0.749 ± 0.164 [−0.866, −0.632]	−0.932 ± 0.166 [−1.051, −0.813]

Data are presented as mean ± SD [95% confidence interval for the mean]. Change values were calculated relative to W1. ROM was included as a manipulation-check variable.

**Table 3:** Linear mixed-model results for the progressive warm-up.

Outcome	Condition Effect	Stage Effect	Condition × Stage	R^2^m	R^2^c	ICC
Acceleration index	F (1, 72) = 1.00; *p* = 0.321	F (3, 72) = 6.12; *p* < 0.001	F (3, 72) = 0.32; *p* = 0.811	0.149	0.488	0.399
Δ time to peak velocity	F (1, 54) < 0.01; *p* = 0.996	F (2, 54) = 9.67; *p* < 0.001	F (2, 54) = 2.17; *p* = 0.124	0.127	0.684	0.638
Δ peak velocity	F (1, 54) = 1.31; *p* = 0.258	F (2, 54) = 75.64; *p* < 0.001	F (2, 54) < 0.01; *p* = 0.999	0.559	0.790	0.524

R^2^m = marginal coefficient of determination; R^2^c = conditional coefficient of determination; ICC = intraclass correlation coefficient. Statistically significant effects are presented in bold. The denominator degrees of freedom reported for the Wald tests are residual degrees of freedom and do not represent participant-level degrees of freedom for the between-participant condition contrast or a cluster-based small-sample correction.

**Table 4:** Descriptive results for the loading protocol.

Outcome	Condition	L1	L2	L3	L4
Acceleration index (ms^−2^)	CAM	5.39 ± 5.23 [1.65, 9.14]	7.35 ± 6.69 [2.57, 12.14]	6.59 ± 6.52 [1.93, 11.26]	8.31 ± 9.23 [1.22, 15.40]
	ST	4.51 ± 5.74 [0.40, 8.62]	6.05 ± 6.44 [1.44, 10.66]	8.15 ± 10.13 [0.90, 15.40]	7.50 ± 7.65 [1.62, 13.38]
First-repetition mean concentric velocity retention (%)	CAM	Reference	107.1 ± 27.1 [87.7, 126.5]	93.9 ± 12.4 [85.0, 102.8]	96.8 ± 10.7 [88.5, 105.0]
	ST	Reference	95.1 ± 12.7 [86.0, 104.2]	89.7 ± 4.2 [86.8, 92.7]	82.9 ± 7.3 [77.3, 88.5]
First-repetition power retention (%)	CAM	Reference	107.8 ± 26.9 [88.5, 127.0]	96.4 ± 10.9 [88.6, 104.2]	97.2 ± 10.9 [88.8, 105.6]
	ST	Reference	94.0 ± 13.1 [84.6, 103.4]	90.4 ± 3.8 [87.7, 93.0]	81.4 ± 6.4 [76.5, 86.4]
Δ time to peak velocity (ms)	CAM	Reference	−1.5 ± 132.4 [−96.2, 93.3]	44.6 ± 224.4 [−115.9, 205.2]	−59.3 ± 235.8 [−240.6, 122.0]
	ST	Reference	−255.6 ± 430.4 [−563.5, 52.3]	−150.8 ± 395.7 [−433.9, 132.3]	−190.4 ± 415.1 [−509.4, 128.7]
Δ peak velocity (ms^−1^)	CAM	Reference	0.040 ± 0.133 [−0.083, 0.162]	0.025 ± 0.119 [−0.074, 0.125]	0.045 ± 0.136 [−0.069, 0.158]
	ST	Reference	−0.012 ± 0.056 [−0.058, 0.034]	−0.032 ± 0.092 [−0.098, 0.034]	−0.073 ± 0.052 [−0.117, −0.030]
Velocity rebound (ms^−1^)	CAM	Reference	0.081 ± 0.051 [0.045, 0.117]	0.049 ± 0.123 [−0.046, 0.143]	0.089 ± 0.063 [0.036, 0.141]
	ST	Reference	0.080 ± 0.076 [0.017, 0.143]	0.110 ± 0.054 [0.065, 0.155]	0.136 ± 0.068 [0.080, 0.193]

Data are presented as mean ± SD [95% confidence interval for the mean]. Retention and change values were calculated relative to L1. For velocity rebound, values presented in the L2, L3, and L4 columns correspond to the L1 → L2, L2 → L3, and L3 → L4 transitions, respectively. Valid data were available for 10 participants per condition at L1–L3 and nine participants per condition at L4. All participants completed the prescribed sets and repetitions. Model-estimated marginal means may differ from the arithmetic averages of the condition-specific raw descriptive means because they are derived from the fitted mixed-effects model using all available repeated observations. This difference may be more apparent at L4 because fewer valid observations were available at this set than at L1–L3.

**Table 5:** Linear mixed-model results for the loading protocol.

Outcome	Condition Effect	Set/Target/ Transition Effect	Interaction	R^2^m	R^2^c	ICC
Acceleration index	F (1, 70) < 0.01; *p* = 0.985	F (3, 70) = 3.23; *p* = 0.027	F (3, 70) = 0.71; *p* = 0.548	0.033	0.790	0.782
First-repetition mean concentric velocity retention	F (1, 52) = 4.56; *p* = 0.038	F (2, 52) = 4.50; *p* = 0.016	F (2, 52) = 0.73; *p* = 0.486	0.203	0.390	0.235
First-repetition power retention	F (1, 52) = 6.94; *p* = 0.011	F (2, 52) = 4.25; *p* = 0.020	F (2, 52) = 0.73; *p* = 0.486	0.235	0.392	0.206
Δ time to peak velocity	F (1, 52) = 4.23; *p* = 0.045	F (2, 52) = 0.37; *p* = 0.694	F (2, 52) = 0.19; *p* = 0.831	0.099	0.199	0.111
Δ peak velocity	F (1, 52) = 2.32; *p* = 0.135	F (2, 52) = 1.07; *p* = 0.353	F (2, 52) = 2.14; *p* = 0.130	0.127	0.811	0.783
Velocity rebound	F (1, 52) = 1.37; *p* = 0.249	F (2, 52) = 2.31; *p* = 0.111	F (2, 52) = 1.82; *p* = 0.174	0.115	0.615	0.565

R^2^m = marginal coefficient of determination; R^2^c = conditional coefficient of determination; ICC = intraclass correlation coefficient. The condition-effect values presented in the table are approximate mixed-model Wald tests. Their denominator degrees of freedom are residual degrees of freedom calculated from the number of valid observations minus the number of estimated fixed-effect coefficients and do not constitute participant-level small-sample inference for the between-participant contrast. Exact participant-level permutation tests were used as the primary inference for the observed between-condition effects in first-repetition mean concentric velocity retention (*p* = 0.044), first-repetition power retention (*p* = 0.012), and change in time to peak velocity (*p* = 0.045).

## Data Availability

The raw data supporting the conclusions of this article will be made available by the author on request.
